# Expression of genes that encode the annexin-1 and galectin-1 proteins in nasal polyposis and their modulation by glucocorticoid

**DOI:** 10.1590/S1808-86942010000200011

**Published:** 2015-10-19

**Authors:** Atílio Maximino Fernandes, Erica Babeto, Paula Rahal, Paola Jocelan Scarin Provazzi, Claudia Augusta Hidalgo, Wilma T. Anselmo-Lima

**Affiliations:** Doctorate, physician of the otorhinolaryngology and head & neck surgery department of Famerp, São José do Rio Preto, SP; Master's degree in genetics, doctoral student in the biology department, Ibilce/UNESP, São José do Rio Preto; Doctorate, adjunct professor of the biology department, Ibilce/UNESP, São José do Rio Preto; Master's degree, doctoral student in the biology department, Ibilce/UNESP, São José do Rio Preto; Doctorate, adjunct professor of mathematics, UNIP, São José do Rio Preto; Associate professor of the ophthalmology, otorhinolaryngology and head & neck surgery department, Ribeirão Preto Medical School, USP

**Keywords:** annexin a1, galectin 1, glucocorticoids, nasal polyps

## Abstract

Rhinosinusal polyps physiopathology is not fully understand, despite numerous hypotheses regarding its inflammatory process.

**Aims:**

a prospective study regarding the gene expression of proteins: anexin-1 and galectin-1, which has an anti-inflammatory action and is modulated by steroids.

**Materials and Methods:**

eleven patients with rhinosinusal polyps suffered a biopsy of their polyps at two moments: in the absence of systemic steroids and during its use. In the two samples we assessed the expression of these genes and compared it to the normal nasal mucosa in the middle meatus.

**Results:**

We noticed that the mean expression of the genes which code anexin-1 and galectin-1 was predominantly increased, regardless of the use of steroids in relation to the control nasal mucosa. Notwithstanding, in polyps without the use of steroids, the mean gene expression of anexin-1 was significantly higher than in the polyps which were under the use of steroids. Regarding galectin-1, there was no significant difference between the expression mean values before and after the use of systemic steroids.

**Conclusion:**

The genes present an expression increase in the polyp mucosa, regardless of the use of steroids; nonetheless, the relationship of these two genes of anti-inflammatory proteins with steroids did not happen the same way.

## INTRODUCTION

Nasal polyps may be characterized by edema of the nasal mucosa associated with degeneration of variable intensity. It generally begins on the middle meatus bilaterally, and may extend to the paranasal sinuses and nasal fossae, probably associated with various clinical etiopathogenic entities.^1^ Recently, nasal polyps as a condition was classified in the chronic rhinosinusitis subgroup, where the common symptoms are nasal congestion and nasal block, facial pain, anterior or posterior discharge, and decreased olfaction; nasal polyps, however, may be differentiated by the presence of bilateral polypoid degeneration in the nasal meatuses.^2^

Lack of a clear definition is due to poor understanding of the etiopathogeny of this condition. It is clear that tissue inflammation with eosinophilia is the main feature of inflammatory and edematous polyps, which comprise about 90% of nasal polyps. However, the mediators of inflammation in this situation are not a conventional inflammatory process; eosinophilic inflammation becomes persistent and unrelated to known mechanisms of atopy. A multifactor cause has been suggested, in which there is a resulting variable degeneration of the nasal mucosa, which results in nasal block, loss of olfaction, and recurring rhinosinusitis.^3^

Animal models have shown that anti-inflammatory proteins, such as annexin-1 and galectin-1, are involved in inflammation. They appear to mediate inflammation when glucocorticoids are administered.^4^, ^5^, ^6^

Annexin-1 is a family of 12 protein found in mammals; these proteins are able to bind to phospholipids and calcium. They are frequent in mammal tissues, with a discreet distribution in certain epithelial cells, such as those of the respiratory and urinary systems, the skin, the synovial, macrophages and tissue leukocytes.^7^ Annexin-1 has been implied in several intracellular processes, such as intracellular signal transduction, cytoskeleton membrane binding, proliferation and differentiation. Its anti-inflammatory function is attributed to its ability to inhibit phospholipase A2 and to bind to specific granulocyte and macrophage surface receptors, inhibiting leukocyte diapedesis.^7^

It has also been demonstrated that glucocorticoids may induce annexin-1 synthesis in monocytes and neutrophils, but not in other cell lineages; in these, glucocorticoid-induced annexin-1 expression is related with cell growth and differentiation.

Galectins are a specific family of lecithins that bind to galactosides. Galectin-1 was the first to be described; it consists of a homodimeric protein, expressed mainly in lymphoid organs such as the thymus, lymph nodes, activated macrophages and T cells. This suggests an important relation with the generation and maintenance immune tolerance. Its exact in vivo function remains uncertain because of rupture of the galectin-1 gene; however, studies with galectin-1 inhibitors in rats have shown inhibition of acute inflammation, probably by a decreased inflow of polymorphonuclear cells.^8^

Immunohistochemical studies involving comparisons between the nasal mucosa of the lower and middle nasal turbinates and nasal polyps and between allergic and non-allergic subjects have shown variable galectin expression in the nasal mucosa. Galectin-1 is expressed more in polyps compared to the middle and lower turbinates; however, a higher expression of galectin-1 has been found in the lower nasal turbinate compared to the middle turbinate. Allergic patients also express more galectin-1, although this difference appears not to be significant.^9^ Such variability raises questions about the possible involvement of galectin-1 in inflammation in nasal polyps. Some authors have questioned whether increased expression of galectin-1 in the lower turbinate, compared to the middle turbinate, would be an anti-inflammatory protective effect with subsequent inhibition of polyp growth; this could explain the absence of polypoid degeneration in the lower turbinate. Increased expression in polyps may be an uncontrolled attempt to block pre-installed inflammation. We therefore decided to investigate the expression of genes that code these two anti-inflammatory proteins (annexin-1 and galectin-1) in nasal polyps and their response when systemic glucocorticoids are given.

## PATIENTS AND METHODS

Nasal polyp specimens were taken from patients with nasal polyps seen at the otorhinolaryngology outpatient unit of the otorhinolaryngology and head & neck surgery department of the São José do Rio Preto medical school (FAMERP) in Sao Paulo. These patients agreed to participate and signed a free informed consent form that had been approved by the Institutional Review Board of the abovementioned institution (process number n^2^ 0220/2001).

This study initially had 15 participants with nasal polyps diagnosed by using nasofibroscopy confirmed at the pathology lab. These patients were also aged over 18 years, and had been referred to surgery because clinical treatment had failed or because complaints of nasal block and anosmia persisted.

Two samples were taken from each of these 15 patients to form two groups of samples. The first group consisted of biopsy samples taken at the first visit, at which point patients were not using systemic, topical or inhaled corticosteroids and/or anti-histaminic drugs; this was group B-1. The second group (B-2) consisted of the biopsies taken during surgery; patients had also been given systemic corticosteroids - a 1 ml ampoule of betamethasone acetate/betamethasone sodium phosphate administered intramuscularly five days before the procedure. The interval between the first and second biopsies varied depending on when the surgery was scheduled; patients were always given corticosteroids before undergoing surgery. Continuous medication for elevated blood pressure or for controlling blood glucose levels was maintained to avoid negative effects on health.

A control group comprised 10 nasal biopsies of the nasal mucosa of the middle meatus of recent cadavers, obtained from the medical examiner's office of the FAMERP; these were patients that died for other reasons, and that had no history of rhinosinusitis or other complaints pertaining to the nose.

All biopsies - taken at the outpatient unit on the first visit (B1), obtained in the surgical theater during surgery (B2), and removed from the nasal mucosa of cadavers (controls) - were promptly placed in a liquid nitrogen tank and then sent to the laboratory for processing to extract RNA with the purpose of verifying the expression of genes coding for the proteins annexin-1 and galectin-1 (RT-QPCR method).^10^

Four patients were taken out of the study sample during RNA extraction, because their sample RNA had degraded. In the end, there were 11 patients with two paired samples, one of patients not using corticosteroids and another of patients using corticosteroids.

The reagent Trizol® (Invitrogen®) was used for extracting RNA. The next step was to establish the concentration of RNA, which was done according to wavelength: DO260/ DO280. Agarose gel electrophoresis (1%) was done of one 1μg of each sample to verify the presence or absence of degraded RNA. Analysis of the gel with ultraviolet light would show an RNA track, with specific 28S and 18S bands representing ribosomal RNA.

From the total extracted RNA, cDNA was obtained by using the cDNA high capacity kit (Applied Biosystems), where 1.25μL anchored oligo (dT) (500μg/mL) and 2μL RNAse Out (40U/μL) are added. The cDNA quality was assessed by PCR amplification of a 613pb fragment of the gene b-ACTIN, which is used as a control for abundant transcripts. The sequences of oligonucleotide bases used for amplifying the fragment were: F 5′ GGCATCGTGATGGACTCCG 3′ and R 5′GCTGGAAGGTGGACAGCG 3′.

Real time quantitative PCR reactions were carried out in the ABI Prism® 7300 real time PCR equipment (Applied Biosystems). Control samples were pooled to minimize variations, since these were samples from different patients.

Sample cDNA was tested three times using the SyberGreen PCR Core Reagent Power Kit (Applied Biosystems). Initiator oligonucleotides for the ANX1 gene were: F 5′ AGAAGATGTATGGTATCTCCCTTTGC 3′ and R 5′ GAGCCACCAGGATTTTCTCATAA 3′. Initiator oligonucleotides for the GAL1 gene were: F 5′ CAGCGGGAGGCTGTCTTTC 3′ and R 5′ CCTGGTCGAAGGTGATGCA 3′. The beta-tubulin gene was used as the endogenous control of the reaction. The oligonucleotide sequence for the beta-tubulin gene was: F 5′ TCAACACCTTCTTCAGTGAAACG 3′ and R 5′ AGTGCCAGTGCGAACTTCATC 3′. The value of the relative expression for the genes Annexin 1 and Galectin 1 were calculated based on Pfaffl's work.^10^

## RESULTS

There were 11 patients whose extracted RNA was of good quality enough to meet the research criteria of this study, of which 8 (72.7%) were male and 3 (27.2%) were female; their age ranged from 35 to 72 years (mean – 53.9 years).

Asthma was present in one subject only (9.0%). Aspirin intolerance was found in two subjects (18.1%). Three subjects had undergone surgery in the past. One subject had systemic arterial hypertension. One subject presented diabetes, and one patient had hypothyroidism. These results are presented in Table 1.

**Table 1:** Clinical features of patients in our sample

Patients	Age	Sex	Aspirin intolerance	Asthma	Previous surgery	Associated conditions
01	68	M	present	–	–	no
02	72	M	–	–	–	Diabetes
03	43	F	–	–	–	no
04	49	M	present	–	–	no
05	53	F	–	–	present	no
07	55	F	–	–	present	Hypothyroidism
08	67	M	–	–	–	Systemic arterial hypertension
09	35	M	–	–	–	no
10	36	M	–	present	–	no
11	52	M	–	–	present	no
13	63	M	–	–	–	Systemic arterial hypertension

An R ratio was applied to demonstrate the gene expression values of the proteins that were studied here; this means the number of times that any specific protein in a specific group was expressed more relative to another group. The R ratio is achieved by processing the same sample three times and calculating the mean of these three reactions; the R ratio can thus be defined as the mean of gene expression that codes a specific protein in three reactions in any given patient. The standard deviation of the mean of three reactions is named DCT DEVIATION. Values of the expression are in log^2^ in the charts to standardize the R values graphically.

An increased gene expression value occurs when the ratio between the study group and the control group is higher than 2 or 1 when using the log^2^ expression; increased expression is not significant when equal to or below this ratio. The same relation applies to negative values - for patients in which gene expression decreased.

**Figure 1:**
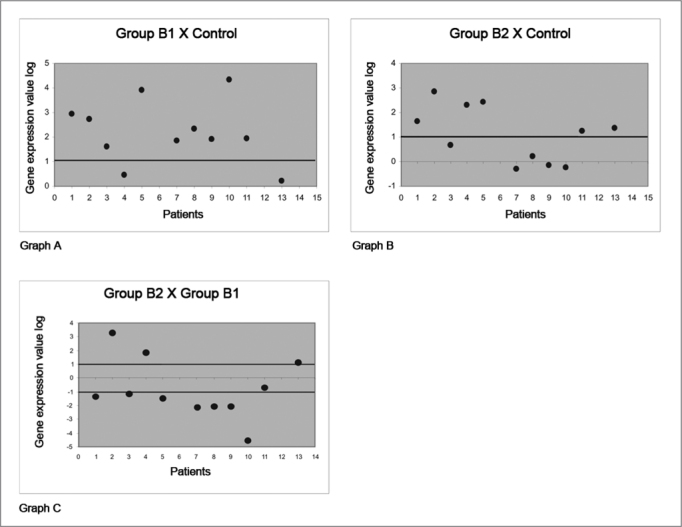
Gene expression values in log2 of the anti-inflammatory protein annexin-1 in nasal polyps. RT-QPCR was used to measure mRNA levels of the annexin-1 protein-coding gene in samples of: normal nasal mucosa - control group; polyps without corticoids - group B1; and polyps with corticoids - group B2. Annexin-1 gene expression in polyp tissues without corticoids compared to controls (Chart A); polyps with corticoids compared to controls (Chart B); and polyps with corticoids compared to polyps without corticoids (Chart C).

Thus, in corticosteroid-free polyps, increased expression of the protein annexin-1 was observed in 9 (81.8%) of 11 subjects compared to nasal mucosa controls (see Chart A of Fig. 1). The analysis of polyps in subjects using corticosteroids, compared to nasal mucosa controls, showed that gene expression was increased in 6 (54.5%) of 11 subjects (see Chart B of Fig. 1). A comparison between polyps of subjects using or not using corticosteroids showed that only 3 subjects (27.2%) had a significantly increased gene expression; however, 7 (63.6%) of 11 subjects had a significantly decreased protein expression (chart C of Fig. 1).

**Figure 2:**
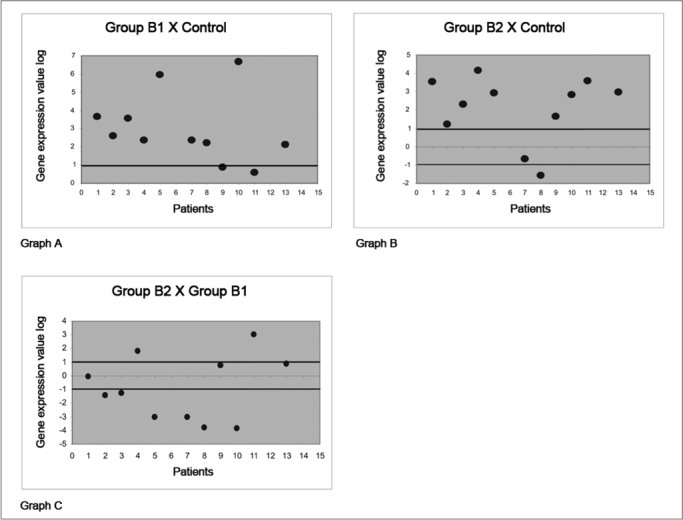
Gene expression values in log 2 of the anti-inflammatory protein galectin-1 in nasal polyps. RT-QPCR was used to measure mRNA levels of the annexin-1 protein-coding gene in samples of: normal nasal mucosa - control group; polyps without corticoids - group B1; and polyps with corticoids - group B2. Galectin-1 gene expression in polyp tissues without corticoids compared to controls (Chart A); polyps with corticoids compared to controls (Chart B); and polyps with corticoids compared to polyps without corticoids (Chart C).

Assessment of the galectin-1 protein comparing corticosteroid-free polyps with nasal mucosa controls showed that expression was increased in 9 (81.9%) of 11 subjects (chart A, Fig. 2). A comparison between polyps of patients using corticosteroids with nasal mucosa controls showed that expression was increased in 9 (81.9%) of 11 subjects (chart B of Fig. 2). A comparison between polyps of subjects using corticosteroids and corticosteroid-free polyps showed that only 3 subjects (27.2%) had a significantly increased galectin-1 gene expression. On the other hand, a significantly decreased expression of the protein galectin-1 was observed in 6 (54.5%) of 11 subjects (chart C of Fig. 2).

## DISCUSSION

The anti-inflammatory effects of annexin-1 has been demonstrated in mice that had their annexin-1 gene suppressed.4 We found that when annexin-1 expression is suppressed, there is an increase in the expression of mRNA for pro-inflammatory cytokines - such as IL-1β, TNF-α, IL-6 and MIF - in synovial cells following induction of inflammation.

Other studies^11^^,^^12^ have shown that antiannexin-1 antibodies block glucocorticoid-induced inhibition of neutrophil and monocyte recruitment. These antibodies also exacerbate inflammation, suggesting an effect of glucocorticoids on inflamed tissues if annexin-1 is also present that enhances its anti-inflammatory effect.

On the other hand, immunohistochemical studies assessing the occurrence of annexin-1 in the nasal mucosa have shown that its expression was altered according to cell type, site, and differentiation. There was, however, no difference in expression between normal and inflamed nasal mucosa, whether in perennial rhinitis or in nasal polyps, suggesting that the expression of annexin-1 remained unaltered in the chronically inflamed nasal mucosa.^7^

By using the RT-QPCR technique, we found that the majority of patients (81.8%) had a significantly increased expression of the gene that codes annexin-1, compared to the normal nasal mucosa. This finding shows that annexin-1 probably is part of inflammation in nasal polyps, since its expression was increased - compared to the normal mucosa - regardless of whether the polyps were under the influence or not of systemic corticosteroids. The mean expression of annexin-1, however, was decreased when subjects were using glucocorticoids; this differs from animal models in which dexamethasone expression increased the expression of the annexin-1 protein.

Annexin-1 increased significantly following administration of glucocorticoids in only two of 11 subjects in this study. The fact that this increased expression did not occur in most subjects appears odd, since annexin is an anti-inflammatory protein. On the other hand, studies of MCF-7 cells that were incubated and treated with dexamethasone or hydrocortisone did not reveal altered expression or cell distribution of annexin.^13^

The effect of annexin appears to occur in the acute phase, during which neutrophils predominate;^11^ the mucosa of nasal polyps, on the other hand, is in a chronic inflammatory state in which eosinophils predominate. The presence of polymorphonuclear cells may vary according to the intensity of inflammation. This seems pertinent to the present study, since the mean expression of annexin-1 was higher in group B1 compared to group B2. Inflammation was more intense in the first group; although still present, inflammation was less intense in the second group because subjects had been given glucocorticoids.

An experimental study of rheumatoid arthritis induced by collagen infiltration6 showed that galectin-1 appears to suppress chronic inflammation; injecting fibroblasts modified to secrete galectin-1 (or continuous administration of recombinant galectin) abolished the clinical and histopathological manifestation of arthritis.

A study that applied immunohistochemical techniques to evaluate the occurrence of galectin-1 in the nasal mucosa found that galectin-1 is expressed more in polyps compared with the mucosa of the middle and inferior nasal turbinate. However, expression tended to be higher in allergic patients and in the lower turbinates, which could be interpreted as protection against inflammation and inhibition of polyp growth, since polyps do not develop in lower turbinates.

In that study, a higher expression of galectin-1 was also found in polyps, compared to the normal nasal mucosa; however, allergic and non-allergic subjects were not grouped separately, since the reported incidence of allergic patients among those with nasal polyps is similar to that of the general population.^14^

It has been reported that the anti-inflammatory effect of glucocorticoids is to increase the transcription of anti-inflammatory genes - such as the leukocyte-inhibitor protein - and to decrease the transcription of inflammation genes,^5^ such as the macrophage colony stimulating factor; such mechanisms, however, remain unclear. Another study evaluated the relation between galectin-1 and the administration of nasal budesonide in patients with nasal polyps;^15^ it showed that budesonide significantly raised galectin-1 expression in the polyps of allergic patients. These results were not seen in non-allergic patients. The study also reported that two allergic patients (out of four) also presented increased expression of the galectin-1 coding gene; expression was decreased in the other two patients, although they had a significantly higher gene expression compared to normal controls.

This study showed that both annexin-1 and galectin-1 are raised in most polyps, regardless of whether patients are using systemic glucocorticoids or not; we found, however, that when glucocorticoids are used, there was a significant decrease in the mean expression of annexin-1 (in the RT-QPCR method). On the other hand, the number of patients with decreased expression of proteins remained the same. The mean expression of galectin-1 was decreased when glucocorticoids were used, but this decrease was not significant, suggesting that the relation between these two anti-inflammatory proteins and glucocorticoids is dissimilar.

We expected initially that corticoids would raise the expression of these anti-inflammatory proteins; we found, however, that polyps without corticoids would present a highly inflamed state, where these proteins are overexpressed, probably to decrease inflammation. When glucocorticoids were given, inflammation decreased, which consequently lowered expression; nevertheless, expression remained high compared to the control nasal mucosa.

## CONCLUSION

We concluded that there is an increased expression of genes that code the anti-inflammatory proteins annexin-1 and galectin-1 in nasal polyps. When subjects were given systemic corticosteroids, the mean gene expression of annexin-1 decreased significantly and the mean expression of galectin-1 remained unaltered.
